# Regulation of α_2B_-Adrenergic Receptor Cell Surface Transport by GGA1 and GGA2

**DOI:** 10.1038/srep37921

**Published:** 2016-11-30

**Authors:** Maoxiang Zhang, Wei Huang, Jie Gao, Alvin V. Terry, Guangyu Wu

**Affiliations:** 1 Department of Pharmacology and Toxicology, Medical College of Georgia, Augusta University, 1459 Laney Walker Blvd., Augusta, GA 30912, USA

## Abstract

The molecular mechanisms that control the targeting of newly synthesized G protein-coupled receptors (GPCRs) to the functional destinations remain poorly elucidated. Here, we have determined the role of Golgi-localized, γ-adaptin ear domain homology, ADP ribosylation factor-binding proteins 1 and 2 (GGA1 and GGA2) in the cell surface transport of α_2B_-adrenergic receptor (α_2B_-AR), a prototypic GPCR, and studied the underlying mechanisms. We demonstrated that knockdown of GGA1 and GGA2 by shRNA and siRNA significantly reduced the cell surface expression of inducibly expressed α_2B_-AR and arrested the receptor in the perinuclear region. Knockdown of each GGA markedly inhibited the dendritic expression of α_2B_-AR in primary cortical neurons. Consistently, depleting GGA1 and GGA2 attenuated receptor-mediated signal transduction measured as ERK1/2 activation and cAMP inhibition. Although full length α_2B_-AR associated with GGA2 but not GGA1, its third intracellular loop was found to directly interact with both GGA1 and GGA2. More interestingly, further mapping of interaction domains showed that the GGA1 hinge region and the GGA2 GAE domain bound to multiple subdomains of the loop. These studies have identified an important function and revealed novel mechanisms of the GGA family proteins in the forward trafficking of a cell surface GPCR.

G protein-coupled receptors (GPCRs) are the largest superfamily of cell surface receptors and their functions are highly regulated by intracellular trafficking processes. As compared with well-characterized internalization, recycling and degradation pathways^1^,^2^,^3^, the molecular mechanisms underlying the cell surface transport of nascent GPCRs from the endoplasmic reticulum (ER) through the Golgi apparatus remain poorly elucidated^4^. Similar to other cell surface proteins such as channels and transporters, GPCR transport to the cell surface has been considered as a constitutive process. However, several studies have suggested that GPCR export to the cell surface can be regulated by extracellular stimuli, mediated through multiple pathways, and in a cell type- and receptor-specific manner^5^,^6^,^7^,^8^. Furthermore, a multitude of regulatory proteins have been identified to enhance the cell surface receptor expression by stabilizing receptor conformation, facilitating receptor maturation and/or promoting receptor delivery to the plasma membrane^9^,^10^,^11^,^12^,^13^,^14^,^15^,^16^. Moreover, recent studies have demonstrated that GPCR export from the ER and the Golgi is dictated by highly conserved motifs^17^,^18^,^19^,^20^,^21^,^22^,^23^. These data suggest that, similar to the endocytic pathway, the anterograde trafficking of GPCRs is a complicated and regulatable cellular process.

Golgi-associated, γ-adaptin homologous, ARF-interacting proteins (GGAs) are well known adaptor proteins for clathrin-coated vesicles. There are three GGA isoforms, namely GGA1, GGA2 and GGA3, in humans which have been well characterized to have similar trafficking function that is to facilitate the transport of cargo proteins from the TGN to the endosomal compartment. All three GGAs have identical domain organizations, containing the N-terminal VHS (the Vps27, Hrs, Stam) domain followed by the GAT (GGAs and TOM1) domain, the hinge region and the C-terminal GAE (γ-adaptin ear) domain. Each domain of GGAs has been shown to interact with specific proteins to coordinate their trafficking functions. Specifically, the N-terminal VHS domain interacts with the DxxLL-type sorting motifs of cargo proteins which cycle between the TGN and the endosomal compartment^24^,^25^,^26^,^27^,^28^,^29^,^30^,^31^,^32^,^33^,^34^,^35^,^36^. These highly coordinated VHS-DxxLL signal interactions specifically sort cargo proteins into the TGN-to-endosome pathway. The GAT domain binds to GTP-bound ARF1 and this interaction, together with PIP4, provide molecular anchors for the recruitment of GGAs onto the TGN. The hinge region interacts with clathrin and this interaction is responsible for the recruitment of clathrin onto the TGN, leading to the formation of clathrin-coated vesicles. The C-terminal GAE domain interacts with a number of accessory proteins regulating GGA-mediated TGN-to-endosome transport^37^,^38^,^39^,^40^,^41^,^42^,^43^,^44^,^45^,^46^.

Our laboratory is interested in dissecting the mechanisms of anterograde trafficking of GPCRs. We have recently demonstrated that GGA3 is required for the TGN-to-cell surface transport of α_2B_-adrenergic receptor (α_2B_-AR), a prototypic member of the GPCR superfamily, and that the function of GGA3 in modulating α_2B_-AR export is mediated through its VHS domain interaction with the receptor, providing the first evidence implicating a role of the GGA family proteins in GPCR trafficking^47^. Here we have expanded these studies to define the role of GGA1 and GGA2 in α_2B_-AR cell surface export and elucidate the underlying mechanisms. We have found that all three GGAs are equally important in regulating the cell surface export of α_2B_-AR and more interestingly, three GGAs physically associate with the receptor via distinct domains. These studies have revealed novel mechanisms of the GGA-mediated cell surface GPCR trafficking.

## Results

### Depletion of GGA1 and GGA2 by shRNA and siRNA attenuates the cell surface transport of inducibly expressed α_2B_-AR

**Figure 1:**
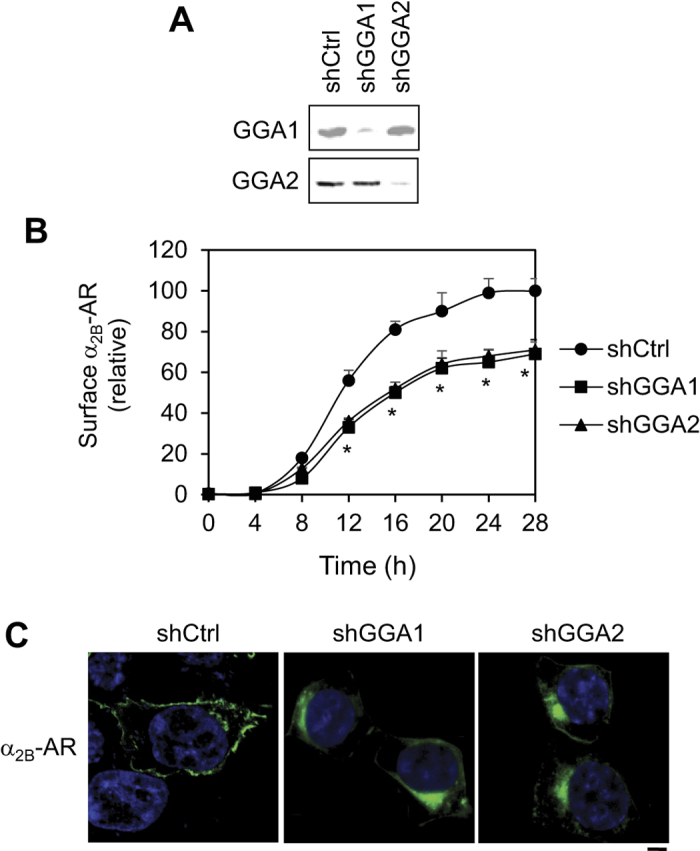
Inhibition of cell surface expression of α_2B_-AR by shRNA-mediated knockdown of GGA1 and GGA2. (**A**) shRNA-mediated depletion of GGA1 and GGA2 in HEK293 cells. The expression of GGAs was measured by immunoblotting using isoform-specific antibodies. (**B**) Effect of shRNA-mediated knockdown of GGA1 and GGA2 on the cell surface expression of α_2B_-AR. HEK293 cells inducibly expressing α_2B_-AR were transfected with control or GGA shRNA and then incubated with doxycycline at the concentration of 40 ng/ml for different time periods (0, 4, 8, 12, 16, 20, 24 and 28 h). The cell surface expression of α_2B_-AR was determined by intact cell ligand binding using [^3^H]-RX821002. The data shown are percentages of specific binding obtained from cells transfected with control shRNA and treated with doxycycline for 28 h, in which the mean value of specific [^3^H]-RX821002 binding was 35,642 ± 985 cpm per well (n = 4) and presented as the mean ± S.E. of at least three individual experiments. *p < 0.05 versus respective control. (**C**) Effect of GGA knockdown on subcellular distribution of α_2B_-AR. HEK293 cells were transiently transfected with α_2B_-AR-GFP together with control or GGA shRNA for 48 h. The subcellular distribution of α_2B_-AR-GFP was revealed by confocal fluorescence microscopy. The data are representative images of at least five separate experiments. Scale bar, 10 μm.

We have generated stable cell lines by using the Tet-On 3G inducible expression system to drive the expression of HA-α_2B_-AR in HEK293 cells and utilized these inducible cells to define the function of GGA3 in the cell surface transport of newly synthesized α_2B_-AR^47^. In the current study, we determined the role of GGA1 and GGA2. HEK293 cells were transfected with previously characterized shRNAs targeting GGA1 and GGA2 (Fig. 1A) and the effect of depleting individual GGAs on the numbers of α_2B_-AR at the cell surface was quantified by intact cell ligand binding assays using the cell nonpermeable radioligand [^3^H]-RX821002 after doxycycline induction for different time periods. shRNA-mediated knockdown of GGA1 and GGA2 similarly inhibited the cell surface expression of α_2B_-AR at each time point and the maximal inhibition was observed by about 30% after doxycycline induction for 20 h (Fig. 1B).

We then determined the effect of GGA1 and GGA2 knockdown on the subcellular distribution of α_2B_-AR. α_2B_-AR was tagged with green fluorescent protein (GFP) and its subcellular distribution was visualized by confocal microscopy in cells transiently transfected with shRNA targeting GGA1 and GGA2. α_2B_-AR was clearly arrested in the perinuclear region, unable to transport to the cell surface, by GGA1 and GGA2 shRNA as compared to control cells in which α_2B_-AR was robustly expressed at the cell surface (Fig. 1C).

**Figure 2:**
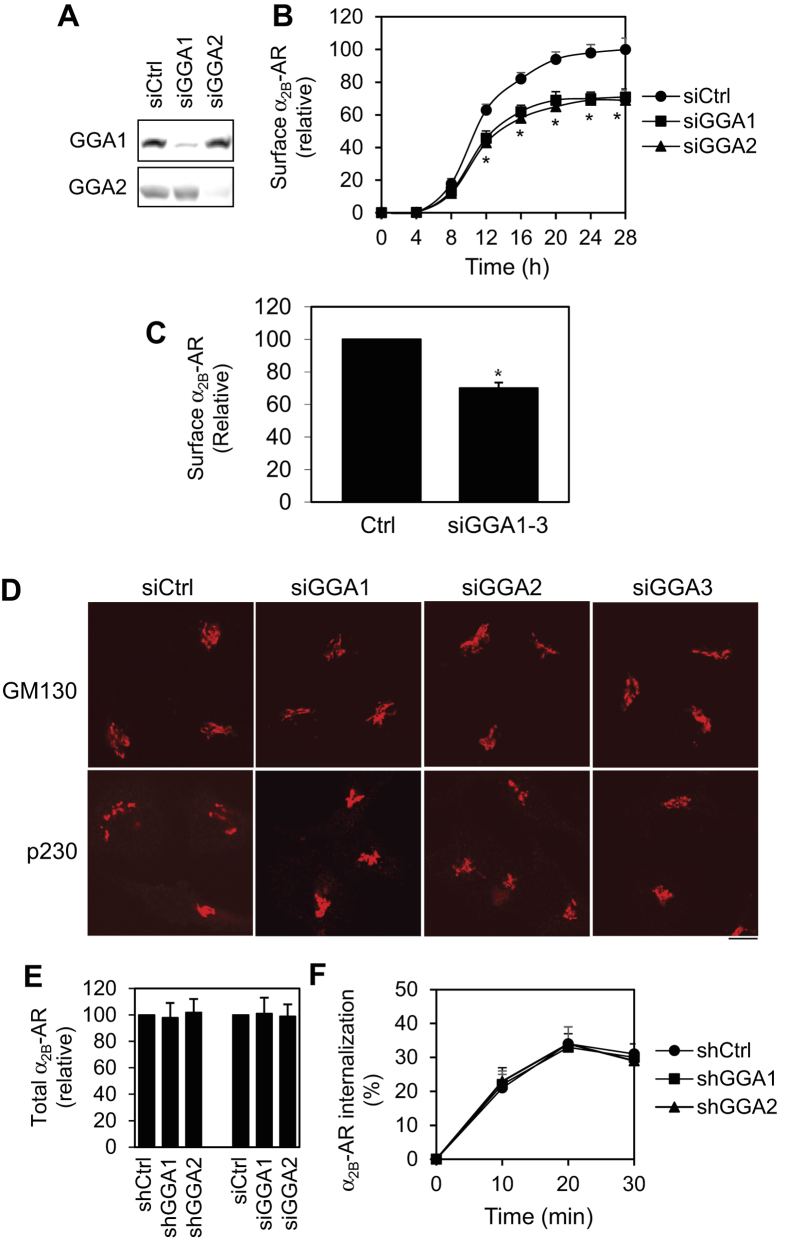
Inhibition of cell surface expression of α_2B_-AR by siRNA-mediated depletion of GGA1 and GGA2. (**A**) siRNA-mediated depletion of GGA1 and GGA2 in HEK293 cells. (**B**) Effect of siRNA-mediated knockdown of GGA1 and GGA2 on the cell surface expression of α_2B_-AR. HEK293 cells inducibly expressing α_2B_-AR were transfected with control siRNA or siRNA targeting GGA1 and GGA2 and incubated with doxycycline as described in legends of Fig. 1B. The average specific binding of [^3^H]-RX821002 from cells without siRNA transfection and treated with doxycycline for 28 h was 34,423 ± 563 cpm per well. (**C**) Effect of combination knockdown of GGA1, GGA2 and GGA3 on the cell surface expression of α_2B_-AR in HEK293 cells. (**D**) Effect of knockdown of GGA1, GGA2 and GGA3 on the Golgi structure. HEK293 cells were transfected with control or GGA siRNA for 48 h and then stained with antibodies against GM130 (1:200 dilution) and p230 (1:100 dilution) overnight. Scale bar, 10 μm. (**E**) Effect of GGA1 and GGA2 knockdown on total α_2B_-AR expression. HEK293 cells inducibly expressing α_2B_-AR were transfected with control or GGA shRNA or siRNA for 24 h and incubated with doxycycline (40 ng/ml) for another 24 h. The overall α_2B_-AR expression was measured by flow cytometry following staining with HA antibodies in permeabilized cells (n = 3). (**F**) Effect of GGA1 and GGA2 knockdown on the internalization of α_2B_-AR. HEK293 cells stably expressing α_2B_-AR were transfected with arrestin-3 and control or GGA shRNA and incubated with doxycycline as described above. The cells were then stimulated with epinephrine (100 μM) for 10, 20 and 30 min (n = 3). The cell surface expression of α_2B_-AR was determined by intact cell ligand binding using [^3^H]-RX821002. The data are presented as the mean ± S.E. of at least three individual experiments in (**B**,**C**,**E**,**F**). *p < 0.05 versus respective control.

We next used the siRNA strategy to deplete GGA1 and GGA2 (Fig. 2A). siRNA-mediated depletion of GGA1 and GGA2 significantly reduced the maximal cell surface expression of inducibly expressed α_2B_-AR (Fig. 2B). Furthermore, simultaneous depletion of GGA1, GGA2 and GGA3 by siRNAs produced a similar inhibitory effect on α_2B_-AR transport as compared to individual GGA knockdown (Fig. 2C). Knockdown of GGAs did not disrupt the general integrity of the Golgi as revealed by confocal microscopy following staining with antibodies against the Golgi marker GM130 and the trans-Golgi network (TGN) marker p230 in HeLa (Fig. 2D) and HEK293 cells (data not shown), suggesting that the reduction of cell surface transport of α_2B_-AR induced by GGA knockdown was unlikely caused by disruption of the Golgi structure which will produce non-specific inhibition on global protein transport. In addition, knockdown of GGA1 and GGA2 by shRNA and siRNA did not affect the overall synthesis of α_2B_-AR as measured by flow cytometry following staining with anti-HA antibodies in permeabilized cells (Fig. 2E). Expression of shRNA targeting GGA1 and GGA2 also did not influence the internalization of α_2B_-AR in response to epinephrine stimulation in HEK293 cells (Fig. 2F), suggesting that reduction of the cell surface expression of α_2B_-AR caused by GGA1 and GGA2 knockdown was not induced by constitutive internalization of the receptor. Altogether, these results demonstrate that both GGA1 and GGA2 are involved in the regulation of cell surface export of α_2B_-AR.

### Depletion of GGA1 and GGA2 inhibits α_2B_-AR expression in the dendrites of cortical neurons

**Figure 3:**
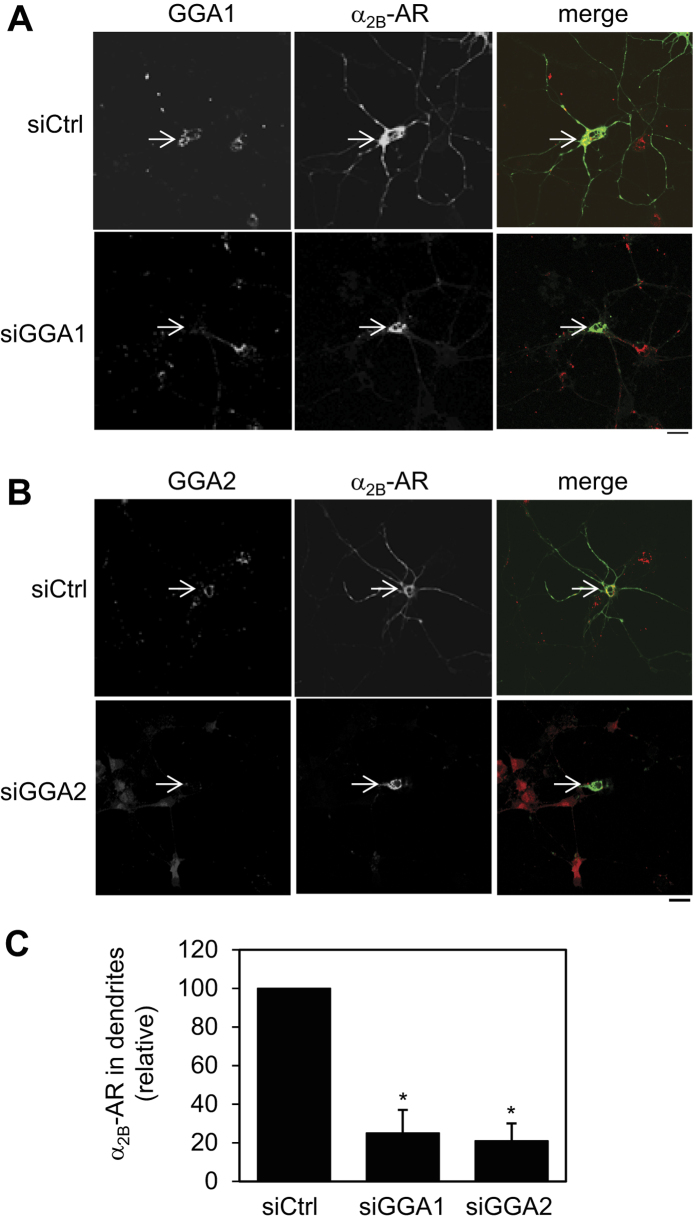
Effect of GGA1 and GGA2 depletion on the dendritic expression of α_2B_-AR in primary cortical neurons. (**A**) Effect of GGA1 knockdown on α_2B_-AR expression in the dendrites of primary cortical neurons. The cortical neurons were transfected with α_2B_-AR-GFP together with GGA1 siRNA at DIV 5. Two days after transfection, the neurons were stained with antibodies against GGA1. The distribution of α_2B_-AR was visualized by confocal microscopy. (**B**) Effect of GGA2 knockdown on the dendritic expression of α_2B_-AR. The data shown are representative images in at least 4 individual experiments. Arrows indicate the expression of GGA1 or GGA2. Scale bars, 20 μm. (**C**) Quantitative data shown in (**A**,**B**) (n = 17). α_2B_-AR expression in the dendrites was determined by measuring the GFP signal. *p < 0.05 versus control.

As α_2B_-AR plays an important role in regulating the sympathetic nervous system, we addressed the question if GGA1 and GGA2 could modulate the transport of α_2B_-AR in the primary cultures of neurons. For this purpose, the cortical neurons were prepared from embryonic rat pups and transfected with α_2B_-AR-GFP together with siRNA targeting individual GGAs. The effect of siRNA-mediated knockdown of GGAs on the expression of α_2B_-AR in the cortical neurons was measured by confocal microscopy. α_2B_-AR-GFP was expressed in the cell body of cortical neurons with or without GGA knockdown. However, its expression in the dendrites was markedly reduced by GGA1 siRNA with an average reduction of 76% as compared with neurons with normal expression of GGA1 (Fig. 3A and C). Knockdown of GGA2 also dramatically reduced the dendritic expression of α_2B_-AR in primary neuronal cultures (Fig. 3B and C).

### Knockdown of GGA1 and GGA2 inhibits α_2B_-AR-mediated signaling

**Figure 4:**
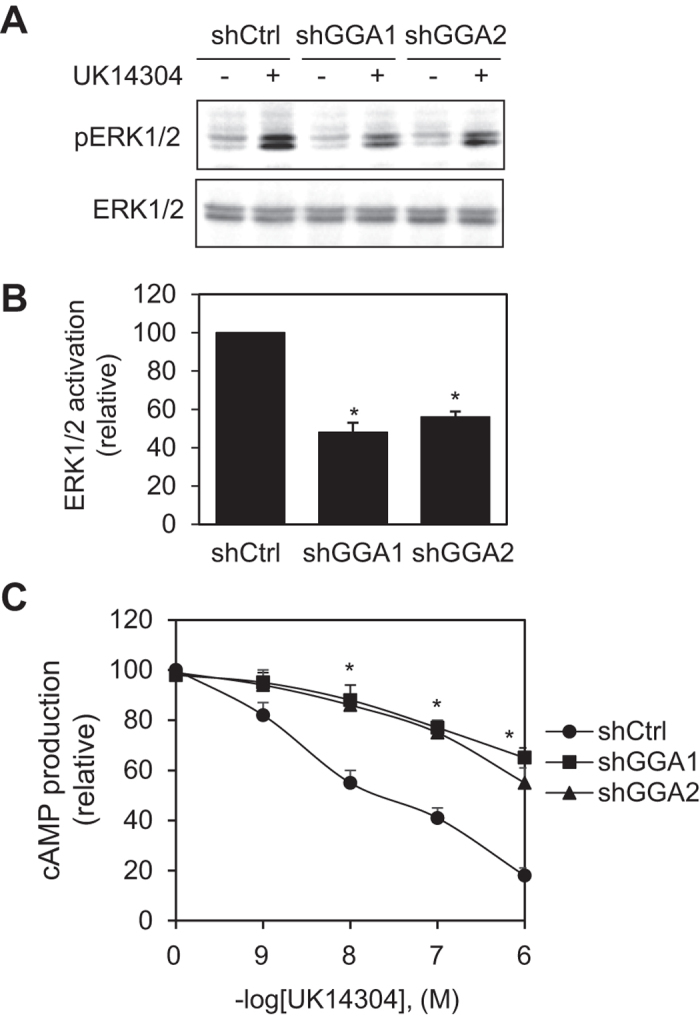
Effect of depleting GGA1 and GGA2 on α_2B_-AR-mediated signaling. (**A**) HEK293 cells were transfected with control shRNA or individual GGA shRNA for 36 h. After starvation for 3 h, the cells were stimulated with UK14304 at the concentration of 1 μM for 5 min at 37 °C. ERK1/2 activation was determined by Western blot analysis using phospho-specific ERK1/2 antibodies. Upper panel is a representative blot of ERK1/2 activation and lower panel shows total ERK1/2 expression. (**B**) Quantitative data shown in A). The data shown are percentages of the mean value obtained from cells transfected with control shRNA and are presented as the mean ± S.E. of at least three experiments. *p < 0.05 versus control shRNA. (**C**) Effect of GGA knockdown on α_2B_-AR-mediated inhibition of cAMP production. HEK293 cells were transfected with α_2B_-AR with or without co-transfection with GGA shRNA and stimulated with forskolin (1 μM) plus different concentrations of UK14304 for 5 min at 37 °C. *p < 0.05 versus respective control.

To determine if GGA-mediated α_2B_-AR trafficking could modulate the function of the receptor, we measured the effect of GGA1 and GGA2 knockdown on the activation of mitogen-activated protein kinases (MAPK) ERK1/2 and the reduction of cAMP production in HEK293 cells. Consistent with the reduction of the cell surface expression of α_2B_-AR, ERK1/2 activation in response to UK14304 stimulation was significantly inhibited by approximately 50% by shRNA targeting GGA1 and GGA2 as compared to cells transfected with control shRNA (Fig. 4A and B). Consistently, shRNA-mediated knockdown of GGA1 and GGA2 reduced α_2B_-AR-mediated inhibition of cAMP production in response to forskolin stimulation (Fig. 4C).

### Differential interaction of GGA1 and GGA2 with α_2B_-AR

**Figure 5:**
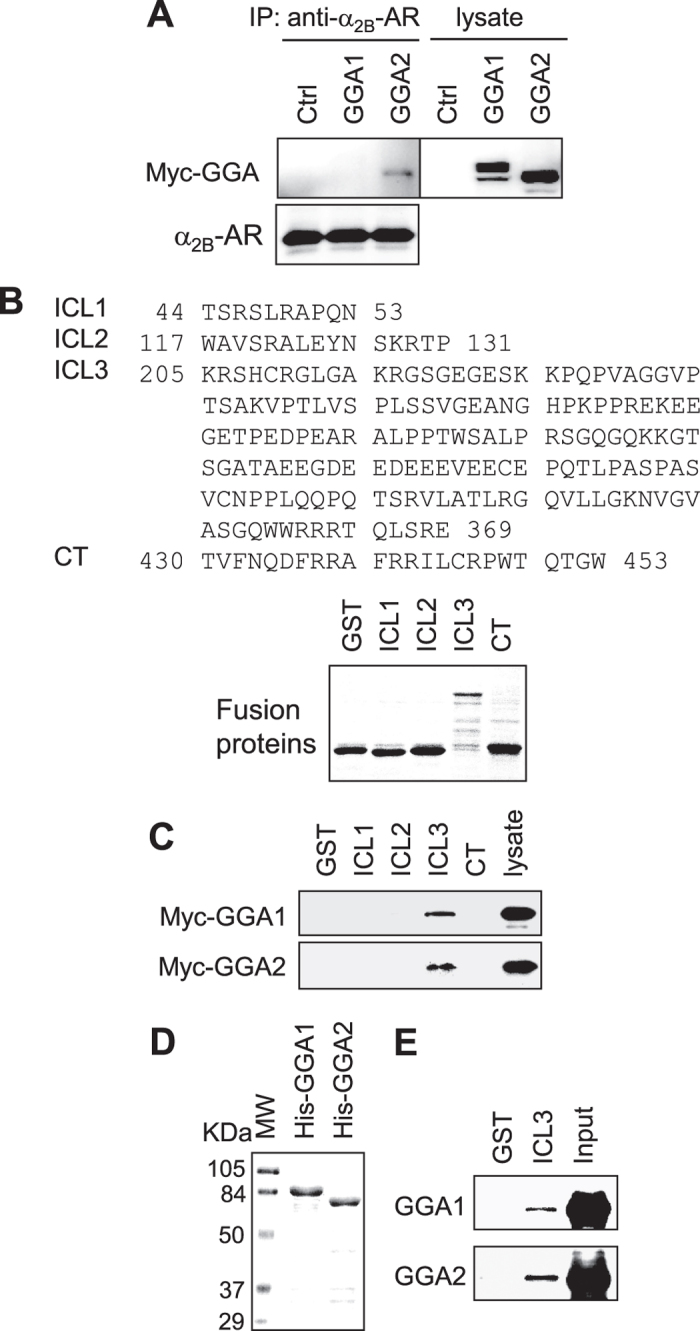
Interaction of α_2B_-AR with GGA1 and GGA2. (**A**) Interaction of α_2B_-AR with GGA1 and GGA2 in co-immunoprecipitation assays. HEK293 cells stably expressing HA-α_2B_-AR were transfected with control vector or myc-tagged GGA1 and GGA2. The receptors were immunoprecipitated with α_2B_-AR antibodies. The amounts of GGA1 and GGA2 (upper panel) and α_2B_-AR (lower panel) were determined by immunoblotting using myc and α_2B_-AR antibodies, respectively. Lysate - 1% of total input. Similar results were obtained in three experiments. (**B**) Sequences of the ICL1, ICL2, ICL3 and C-terminus (CT) of α_2B_-AR (upper panel) and Coomassie blue staining of purified GST fusion proteins (low panel). The calculated molecular weights of GST and the ICL1, ICL2, ICL3, and CT GST fusion proteins are 27,898, 27,422, 28,070, 43,779 and 29,348 daltons, respectively. (**C**) Interaction of different intracellular domains of α_2B_-AR with GGA1 and GGA2. Myc-tagged GGA1 and GGA2 were expressed in HEK293 cells and total cell homogenates were incubated with GST fusion proteins. Bound GGAs were revealed by immunoblotting using anti-myc antibodies. (**D**) Purified His-tagged GGA1 and GGA2. The molecular weight (MW) markers (KDa) are indicated on the left. (**E**) Direct interaction of the α_2B_-AR ICL3 with GGA1 and GGA2. Purified His-tagged GGA1 and GGA2 were incubated with GST-ICL3 fusion proteins and bound GGAs were detected by immunoblotting using anti-His antibodies. Similar results were obtained in at least three separate experiments. Lysate −5% of total input. Similar results were obtained in at least 3 experiments.

We have previously shown that GGA3 interacts with α_2B_-AR and the interaction is mediated through the VHS domain of GGA3 and the third intracellular loop (ICL3) of the receptor^47^. To elucidate the possible molecular mechanisms underlying the function of GGA1 and GGA2 in α_2B_-AR export, we determined if GGA1 and GGA2 could interact with the receptor in co-immunoprecipitation and GST fusion protein pulldown assays. HEK293 cells stably expressing α_2B_-AR were transiently transfected with myc-GGA1 or myc-GGA2 followed by immunoprecipitation using α_2B_-AR antibodies. GGA2 was clearly detected in the immunoprecipitates of α_2B_-AR antibodies, whereas GGA1 was undetectable in the immunoprecipitates (Fig. 5A).

In GST fusion protein pulldown assays, the ICL1, the ICL2, the ICL3 and the C-terminus of α_2B_-AR were generated as GST fusion proteins (Fig. 5B) and incubated with cell lysates expressing myc-GGA1 or myc-GGA2. The GST-ICL3 strongly interacted with GGA2, whereas the ICL1, ICL2 and C-terminus GST fusion proteins did not (Fig. 5C). Interestingly, although GGA1 did not associate with α_2B_-AR in co-immunoprecipitation assays, it interacted with the ICL3, but not the ICL1, the ICL2 and the C-terminus in GST fusion protein pulldown assays (Fig. 5C).

We next determined if GGA1 and GGA2 were able to directly interact with the α_2B_-AR ICL3. In this experiment, GGA1 and GGA2 were tagged with the epitope His and purified (Fig. 5D). GST-ICL3 fusion proteins, but not GST alone, bound to His-tagged GGA1 and GGA2 in GST fusion protein pulldown assays (Fig. 5E). These data indicate that the α_2B_-AR ICL3 interaction with GGA1 and GGA2 is direct. These data also suggest that both GGA1 and GGA2 interact the α_2B_-AR ICL3 with comparable efficiencies.

### Identification of interaction domains of GGAs and α_2B_-AR

**Figure 6:**
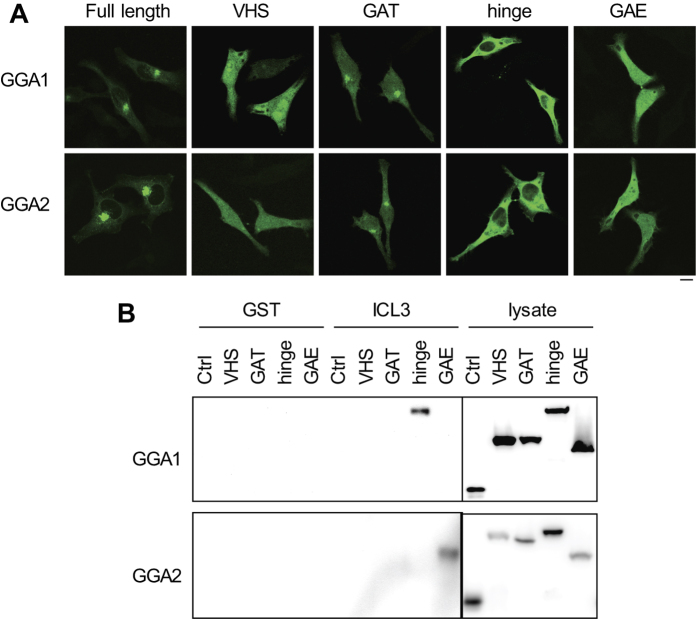
Identification of the α_2B_-AR-binding sites in GGA1 and GGA2. (**A**) Subcellular distribution of GGA1 and GGA2 and their domains revealed by confocal microscopy. HeLa cells were transfected with GFP-tagged GGA1, GGA2 or individual domains for 24 h. Similar results were obtained in at least three separate experiments. Scale bar, 10 μm. (**B**) Interaction of different domains of GGA1 and GGA2 with GST-ICL3. Amino acid sequence analyses showed that the identities of the VHS, GAT, hinge and GAE domains between GGA1 and GGA2 are approximately 65, 61, 26 and 55%, respectively. The GFP-tagged VHS, GAT, hinge and GAE domains of GGA1 and GGA2 were expressed in HEK293 cells. Total cell lysates were incubated with GST-ICL3 fusion proteins. Bound GGA domains were revealed by immunoblotting using GFP antibodies. Total cell lysates expressing GFP alone were used as a control. Lysate – 5% of total input. Similar results were obtained in at least three different experiments.

To define the domains of GGA1 and GGA2 interacting with α_2B_-AR, their VHS, GAT, hinge and GAE domains were tagged with GFP. Confocal microscopy showed that full length GGA1 and GGA2 and their GAT domains were mainly localized to the Golgi whereas the hinge, the VHS and GAE domains were largely expressed in the cytoplasm. In addition, the VHS and GAE domains were also found to be partially expressed in the nuclear compartment (Fig. 6A). The GGA1 hinge domain, but not the VHS, GAT and GAE domains, strongly bound to GST-ICL3 fusion proteins, whereas the GGA2 GAE domain, but not the VHS, the GAT and the hinge domains, was found to interact with the ICL3 of α_2B_-AR (Fig. 6B). These data demonstrate that the α_2B_-AR ICL3 specifically interacts with the hinge domain of GGA1 and the GAE domain of GGA2.

**Figure 7:**
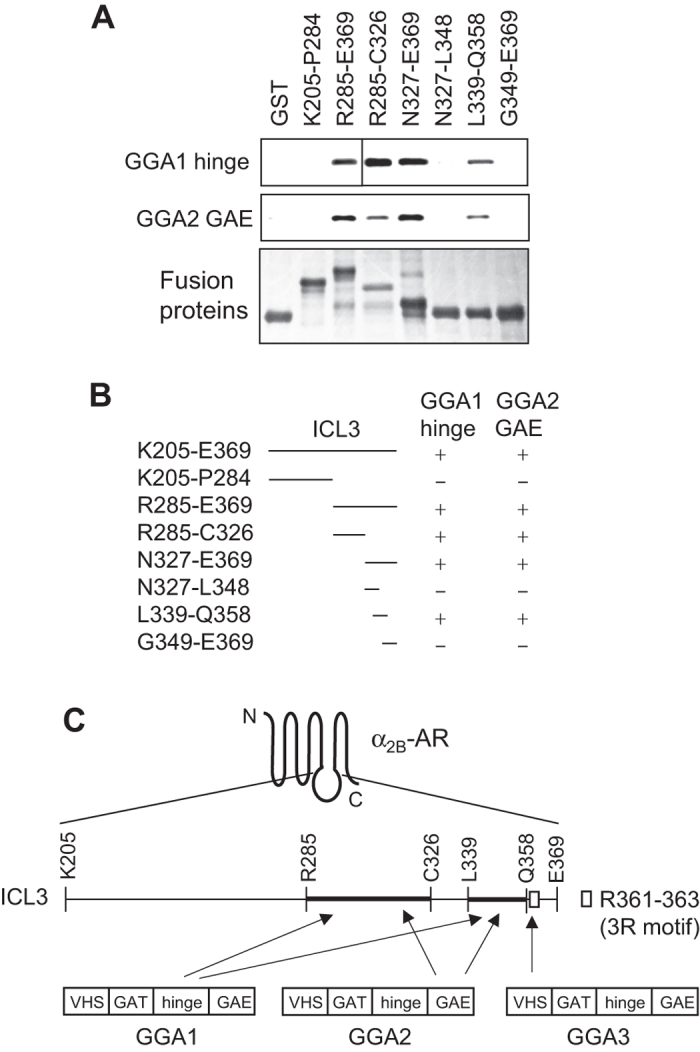
Identification of the GGA1- and GGA2-binding sites in the α_2B_-AR ICL3 by progressive deletion. (**A**) Interaction of different ICL3 fragments with the GGA1 hinge and the GGA2 GAE domains. Each ICL3 fragment was generated as GST fusion proteins. The GGA1 hinge and the GGA2 GAE domains were generated as GFP fusion proteins. Their interactions were determined in GST fusion protein pulldown assays. Bound GGA domains were revealed by immunoblotting using GFP antibodies. Bottom panel shows Coomassie blue staining of purified GST fusion proteins. Similar results were obtained in at least three different experiments. The blots from two gels that were run under the same experimental conditions were combined to show the interaction of the GGA1 hinge with different ICL3 domains (upper panel). (**B**) A summary of progressive deletion to identify the GGA1- and GGA2-binding domains in the α_2B_-AR ICL3 as shown in (**A**). +Interacting with individual GGA domains; −, not interacting with GGA. (**C**) A diagram showing differential interactions between α_2B_-AR and three GGAs. The GGA1 hinge and the GGA2 GAE domains bind to two subdomains of the α_2B_-AR ICL3 as revealed in the current studies, whereas the GGA3 VHS domain interacts with the α_2B_-AR ICL3, specifically the 3R motif, as demonstrated in our previous studies^47^.

To further map the GGA1 and GGA2-binding sites in the α_2B_-AR ICL3, the ICL3 was progressively deleted and generated as GST fusion proteins. The C-terminal portion R285-E369 strongly interacted with the GGA1 hinge and the GGA2 GAE whereas the N-terminal portion K205-P284 did not (Fig. 7A). Furthermore, the GST fusion proteins encoding R285-C326, N327-E369 and L339-Q358 interacted with the GGA1 hinge domain and the GGA2 GAE domain whereas the fragments N327-L348 and G349-369 did not (Fig. 7A). These data demonstrate that there are two binding sites for GGA1 and GGA2, one located in the region R285-C326 and the other in the region L339-Q358 (Fig. 7B).

## Discussion

Three GGA family proteins are well-characterized adaptor proteins for clathrin-coated vesicles that transport cargo proteins specifically from the TGN to the endosomal compartment. We have recently demonstrated that GGA3 depletion attenuated the cell surface transport of α_2B_-AR and arrested the receptor in the Golgi/TGN compartment^47^. These data provide the first direct evidence implicating a role for the GGA family proteins in controlling the cell surface GPCR transport. Our current studies have shown that, similar to GGA3, knockdown of GGA1 and GGA2 significantly reduced the cell surface expression of α_2B_-AR in cells and primary neurons as quantified by intact live cell ligand binding and direct visualization of receptor subcellular localization. These data indicate that all three GGAs are involved in the anterograde cell surface traffic of α_2B_-AR.

There are several interesting points regarding the regulation of cell surface transport of α_2B_-AR by the GGA family proteins. First, as depleting GGA1, GGA2, and GGA3 individually or in combination similarly inhibited the cell surface transport of α_2B_-AR, three GGAs are equally important in mediating the export of newly synthesized receptor These data also suggest that the anterograde transport of α_2B_-AR requires all three GGAs and the lack of any one GGA will disrupt the transport. Second, inhibitory effects on the cell surface α_2B_-AR export caused by depleting individual GGAs were moderate (less than 40%). One possible explanation for this phenomenon is that there are multiple pathways to direct α_2B_-AR export trafficking and GGAs mediate only one of these pathways. It should be pointed out that the data described here do not provide sufficient evidence indicating that α_2B_-AR transport from the Golgi/TGN to the cell surface is direct. As proteins destined for the plasma membrane can be transported either directly from the Golgi to the plasma membrane or from the Golgi through the recycling endosomal compartment to the plasma membrane^48^,^49^, it is possible that α_2B_-AR targeting to the cell surface passes through the recycling endosomal compartment. In support of this possibility, GGA3 was shown to modulate the transport of internalized Met receptor tyrosine kinase from the recycling endosomes^50^. Third, GGA knockdown markedly inhibited the expression of α_2B_-AR in the dendrites of primary cortical neurons, implicating that GGAs may play a more important role in the dendritic transport of α_2B_-AR in the native neurons. Fourth, consistent with the reduction of cell surface receptor expression, knockdown of individual GGAs suppressed α_2B_-AR-mediated signaling measured as ERK1/2 activation and cAMP reduction, suggesting that GGAs modulate not only the cell surface trafficking but also the function of the receptor.

Another important finding presented here is that we have elucidated novel mechanisms underlying the function of the GGA family proteins in the cell surface transport of α_2B_-AR. It has been well described that the function of GGAs in sorting proteins into the TGN-to-endosome pathway is tightly controlled by their VHS domain interaction with the DxxLL-type motifs of cargo proteins. These proteins include cation-dependent and cation-independent mannose 6-phosphate receptors, sortilin, sorting-protein-related receptor, low-density lipoprotein receptor-related proteins and β-secretase^24^,^25^,^26^,^27^,^28^,^29^,^30^,^31^,^32^,^33^,^34^. In addition to cargo proteins, many accessory proteins that regulate GGA-mediated trafficking processes also physically associate with GGAs and the sequences D/EFGXØ have been identified as specific GAE-binding motifs in several proteins, including rabaptin-5, p56 and gamma-synergin^51^. We previously showed that the GGA3 VHS domain interacted with the ICL3 of α_2B_-AR. Here we found that GGA1 and GGA2 also interacted with the ICL3 of α_2B_-AR in GST fusion protein pulldown assays. Although the VHS domains are highly conserved amongst three GGAs, the VHS domains of GGA1 and GGA2 did not interact with α_2B_-AR. Interestingly, the α_2B_-AR-binding domains were identified as the hinge domain of GGA1 and the GAE domain of GGA2 domain. Although our data have clearly shown that GGA2 and GGA3 were able to form complexes with α_2B_-AR in co-immunoprecipitation assays, GGA1 did not under the same experimental condition, suggesting that interactions of three GGAs with α_2B_-AR have different regulatory properties. Nevertheless, our previous and current studies indicate that the GGA1 hinge domain, the GGA2 GAE domain and the GGA3 VHS domain are responsible for the interaction with α_2B_-AR (Fig. 7C). These data suggest that the interaction of α_2B_-AR with each GGA is highly specific. To the best of our knowledge, α_2B_-AR is the only cargo molecule identified thus far which is able to interact with distinct domains of three GGAs (Fig. 7C).

Consistent with the identification of different domains in three GGAs responsible for interaction with α_2B_-AR, different GGAs may have different binding sites in the ICL3 of α_2B_-AR. We have previously shown that the 3 R motif in the ICL3 of the receptor and the acidic motif EDWE located in the VHS domain of GGA3 are responsible for the interaction between the receptor and GGA3^47^. Consistent with the fact that the VHS domains of GGA1 and GGA2 did not bind to α_2B_-AR, or the α_2B_-AR ICL3, an alignment of three GGA VHS domains showed that the GGA1 and GGA2 do not have the GGA3-binding motif EDWE, but instead have the sequences LDWA and QDWS, respectively (data not shown). We have used the progressive deletion strategy to successfully map the binding sites of both GGA1 and GGA2 to the regions R285-C326 and L339-Q358. As the GGA-binding subdomains R285-C326 and L339-Q358 do not possess the D/EFGXØ and DxxLL-type motifs and thus, they may contain novel, yet unidentified, GGA-binding signals. Our unpublished data showed that deletion of the fragment R285-Q358 significantly reduced the interaction of α_2B_-AR with GGA2 in coimmunoprecipitation assays. However, the truncated receptor was unable to export to the cell surface. These data suggest that, in addition to binding to GGAs, the ICL3 fragment R285-Q358 may contain other signals important for receptor transport to the cell surface. Nevertheless, these data strongly demonstrate differential interactions of three GGAs with α_2B_-AR which are mediated through different domains/motifs in individual GGAs and the receptor (Fig. 7C).

Similar to α_2B_-AR, many GPCRs possess a large ICL3. In addition to heterotrimeric G proteins coupled to the receptors, a number of ICL3-interacting proteins have been identified and described to play a crucial role in regulating the phosphorylation, trafficking and signal initiation, propagation and termination of the receptors^52^,^53^,^54^,^55^,^56^,^57^,^58^,^59^. Specifically, the α_2B_-AR ICL3 interacts with arrestins, kinases, 14-3-3, spinophilin, the ubiquitin carboxyl terminal esterase L1, ADP ribosylation factor 1 and Sec24C/D^60^,^61^,^62^,^63^,^64^,^65^,^66^,^67^. Our previous report^47^ and current studies have demonstrated that α_2B_-AR uses its ICL3 as a docking site for multiple GGAs. It is interesting to note that different proteins may bind to different ICL3 regions. For example, spinophilin binding sites were mapped to the extreme N-terminal and C-terminal ends of the ICL3 of α_2A_-AR, whereas the C-terminal portions of the loop is important for arrestin interaction^68^. The identification of two subdomains in the ICL3 which are capable of binding to GGA1 and GGA2 suggests multiple contacting points between α_2B_-AR and GGA1 or GGA2. These data also imply that GGAs may interact with a specific three dimensional surface of the loop and these specific interactions form a unique transport machinery that drives forward trafficking of the receptor from the Golgi/TGN to the cell surface.

It has become increasingly apparent that mistrafficking of GPCRs which leads to the dysfunction of the receptors directly links to pathogenesis of human diseases, such as nephrogenic diabetes insipidus, retinitis pigmentosa and male pseudohermaphroditism^69^,^70^,^71^. However, the molecular mechanisms of anterograde transport of the GPCR superfamily to their functional destinations are poorly understood. Our previous studies have identified several highly conserved motifs and regulatory proteins that are required for the cell surface export of α_2B_-AR, as well as other GPCRs, *en route* from the ER and the Golgi^17^,^65^,^72^,^73^,^74^,^75^,^76^. Our previous and current studies have also clearly demonstrated an important role of the GGA family proteins in the cell surface targeting of nascent α_2B_-AR which is likely mediated through physical interactions. In addition to α_2B_-AR, GGA3 was shown to regulate the transport of α_2C_-AR, but not α_2A_-AR, suggesting that there is a specificity of GGA3 for different GPCRs^47^. As the GGA3-binding motif of α_2B_-AR is highly conserved in many GPCRs, such as some muscarinic and serotonin receptor types^47^,^65^. GGA3 may regulate the cell surface transport of a group of GPCRs. However, it still remains unknown if GGA1 and GGA2 are involved in the cell surface export of other GPCRs. To further elucidate the function of the GGA family proteins in the trafficking of the GPCR superfamily will enhance our understanding of GPCR targeting process and may be used to design novel therapeutics for effective therapy of human diseases, involving abnormal trafficking and signaling of GPCRs.

## Materials and Methods

### Materials

Full length GGA1 and GGA2 tagged with myc at their N-termini were generously provided by Dr. Juan S. Bonifacino (Eunice Kennedy Shriver National Institute of Child Health and Human Development, NIH). Arrestin-3 was obtained from Dr. Jeffrey L. Benovic (Thomas Jefferson University). Antibodies against GGA1 were purchased from Abcam Inc. (Cambridge, MA). Antibodies against GGA2, GM130 and p230 were from BD Transduction Laboratories (San Diego, CA). Antibodies against GFP, myc and phospho-ERK1/2 were obtained from Santa Cruz Biotechnology, Inc. (Santa Cruz, CA). Anti-ERK antibodies detecting total ERK1/2 expression and GM130 were from Cell Signaling Technology, Inc. (Beverly, MA). Alexa Fluor 594-labeled antibodies were from Molecular Probes, Inc. (Eugene, OR). Anti-His antibodies and UK14304 were obtained from Sigma-Aldrich (St. Louis, MO). [^3^H]-RX821002 (41 Ci/mmol) was purchased from Perkin Elmer Life Sciences (Waltham, MA). All other materials were obtained as described elsewhere^47^,^65^,^73^.

### Plasmid constructions

α_2B_-AR tagged with either GFP at its C-terminus in the pEGFP-N1 vector or three HA (YPYDVPDYA) at its N-terminus in the pcDNA3.1 (−) vector were generated as described previously^65^. The GST fusion protein constructs coding the first (ICL1, 44–53 residues), the second (ICL2, 117–131 residues), and the third intracellular loops (ICL3, 205–369 residues), different lengths of the ICL3 (K205-P284, R285-E369, R285-C326, N327-E369, N327-L348, L339-Q359 and G349-E369), and the C-terminus (430–453 residues) of α_2B_-AR were cloned into the BamH1 and XhoI restriction sites of the pGEX-4T-1 vector and a stop codon was added before the XhoI restriction site as described previously^65^,^74^. To generate GGA1 domains (VHS: 1–150 residues, GAT: 151–302 residues, hinge: 303–513 residues and GAE, 514–639 residues) and GGA2 domains (VHS: 1–163 residues, GAT: 164–315 residues, hinge: 316–483 residues and GAE, 484–613 residues), each domain was generated by PCR and then cloned into the pEGFP-C1 vector. The sequence of each construct used in this study was verified by restriction mapping and nucleotide sequence analysis.

### Cell culture, primary neuronal preparation and transient transfection

HEK293 and HeLa cells were cultured in Dulbecco’s Modified Eagle’s medium (DMEM) with 10% fetal bovine serum, 100 units/ml penicillin, and 100 μg/ml streptomycin. Transient transfection of cells was carried out using Lipofectamine 2000 reagent (Invitrogen) as described previously^73^. The transfection efficiency was estimated to be greater than 70% based on the GFP fluorescence. Neuronal cultures were prepared from the cortex of embryonic day 18 rat pups and grown on glass coverslips precoated with poly-L-lysine. After 4–5 days *in vitro* (DIV), the neurons were transfected with α_2B_-AR-GFP with or without co-transfection with GGA siRNA by Lipofectamine 2000. The use and care of animals used in this study follows the guidelines of the Augusta University Institutional Animal Care & Use Committee (IACUC). The preparation of primary neurons from timed-pregnant rats was approved by the Augusta University IACUC.

### Generation of inducible cell lines expressing α_2B_-AR

The Tet-On 3 G Tetracycline Inducible Gene Expression System (Clontech Laboratories, Inc.) was utilized to generate stable cell lines inducibly expressing HA-α_2B_-AR in HEK293 cells as described previously^47^. Intact cell ligand binding assays, immunoblotting and confocal microscopy were used to characterize inducible expression of α_2B_-AR at the cell surface^47^. A cell line expressing 8.5 × 10^5^ α_2B_-AR per cell after incubation with doxycycline at a concentration of 40 ng/ml for 24 h was utilized in the current study.

### shRNA- and siRNA-mediated depletion of GGAs

shRNA targeting GGA1 (463AAGCTTCCAGATGACACTACC483) and GGA2 (1428AATACACCTCTGGCTCAAGTG1448) were kindly provided by Dr. Stuart Kornfeld (Washington University School of Medicine) as described^35^. For shRNA-mediated knockdown of GGA1 and GGA2, cells cultured on 6-well plates were transiently transfected with 2.0 μg of control shRNA or shRNA targeting individual GGAs for 24 h. The cells were split into 12 wells at a density of 5 × 10^5^ cells per well and cultured for additional 24 h before measuring the cell surface expression of α_2B_-AR in intact cell ligand binding assays. For siRNA-mediated knockdown of GGAs, siRNAs targeting GGA1 (173CACAGGAGTGGGAGGCGAT191), GGA2 (1291TGAATTATGTTTCGCAGAA1310) and GGA3 (1703 TGTGACAGCCTACGATAAA 1721) and a negative control med GC duplex were purchased from Invitrogen. HEK293 cells were cultured in 6-well dishes at a density of 1 × 10^6 ^cells/well for 24 h and transfected with control or GGA siRNA. The expression of GGA1 and GGA2 was measured by GGA isoform-specific antibodies.

### Measurement of the cell surface and total expression of α_2B_-AR

The cell surface expression of α_2B_-AR was measured by ligand binding of intact live cells using [^3^H]-RX821002 as described^65^,^74^. Briefly, inducible HEK293 cells expressing α_2B_-AR were cultured on 6-well dishes and transiently transfected as described above for 12 h. The cells were split into 12-well plates and cultured for an additional 24 h. After induction with doxycycline at a concentration of 40 ng/ml for different time periods, the cells were incubated with DMEM plus [^3^H]-RX821002 at a concentration of 20 nM in a total volume of 400 μl for 90 min at room temperature. The non-specific binding of α_2B_-AR was determined in the presence of rauwolscine (10 μM). The binding was terminated and excess radioligand eliminated by washing the cells with ice-cold DMEM. The retained radioligands were then extracted by digesting the cells in 1 M NaOH for 2 h. The radioactivity was counted by liquid scintillation spectrometry. All radioligand binding assays were performed in triplicate. For measurement of α_2B_-AR internalization, HEK293 cells stably expressing α_2B_-AR were cultured on 6-well dishes and transfected with control or GGA shRNA together with 1 μg of arrestin-3 for 24 h. After starvation for 3 h, the cells were stimulated with epinephrine at a concentration of 100 μM for different time periods. The cells were washed three times with cold phosphate buffered saline (PBS) and α_2B_-AR expression at the cell surface was measured by intact cell ligand binding at 4 °C as described above.

Total α_2_-AR expression was measured by flow cytometry as described previously^75^. Briefly, HEK293 cells expressing HA-α_2B_-AR were suspended in PBS containing 1% fetal calf serum at a density of 4 × 10^6 ^cells/ml and permeabilized with 0.2% Triton X-100 in PBS for 5 min on ice. The cells were then incubated with high affinity anti-HA-fluorescein (3F10) at a final concentration of 2 μg/ml at 4 °C for 30 min. After washing with 0.5 ml of PBS twice, the cells were resuspended and the fluorescence was analyzed on a flow cytometer (Dickinson FACSCalibur).

### Fluorescence microscopy

Fluorescence microscopic analysis of the subcellular localization of α_2B_-AR was carried out as described previously^73^. Briefly, cells were grown on coverslips precoated with poly-L-lysine in 6-well plates and transfected with 50 ng of α_2B_-AR-GFP together with 400 ng of GGA shRNA. The coverslips were mounted with prolong antifade reagent (Invitrogen) and images were captured using a LSM510 Zessis confocal microscope equipped with a 63× objective. To visualize the localization of α_2B_-AR in primary neurons, the neuronal cultures were transfected with α_2B_-AR-GFP with or without co-transfection with GGA siRNA for 2 days. The neurons were fixed, permeabilized and stained with GGA-isoform specific antibodies at a dilution of 1:500. The amounts of α_2B_-AR-GFP signal pixels in the dendrites were determined by using NIH Image J software as described previously^76^.

### Measurement of ERK1/2 activation

HEK293 cells were cultured on 6-well dishes and transfected with 2 μg of GGA shRNA as described above. The cells were starved for at least 3 h before stimulation with 1 μM UK14304 for 5 min. Stimulation was terminated by addition of 1 × SDS-loading buffer. After solubilizing the cells, 20 μl of total cell lysates were separated by 12% SDS-PAGE. ERK1/2 activation was determined by measuring the levels of phosphorylation of ERK1/2 with phospho-specific ERK1/2 antibodies by immunoblotting^73^.

### Measurement of cAMP production

cAMP concentrations were measured by using the cAMP Direct Biotrak Enzyme Immunoassay System (GE Healthcare Life Sciences) as described previously^76^. Briefly, HEK293 cells were cultured in 6-well dishes and transfected with 0.5 μg of α_2B_-AR with or without GGA shRNA. The cells were then split into 96-well plates at a density of 1 × 10^4 ^cells/well. After starvation for 1 h, the cells were stimulated with forskolin (1 μM) plus or minus different concentrations of UK14304 (1 to 1000 nM) in the presence of 0.1 mM isobutylmethylxanthine for 5 min at 37 °C. The reactions were stopped by aspirating the medium and the cells were lysed with 100 μl of lysis buffer. 50 μl of cell lysate was transferred into microtitre plates and cAMP concentrations were measured according to the protocol provided by the Kit.

### Co-immunoprecipitation

HEK293 cells inducibly expressing HA-α_2B_-AR were cultured on 100-mm dishes and transfected with 10 μg of myc-GGA1 or myc-GGA2 for 24 h. After incubation with doxycycline (40 ng/ml) for 24 h, the cells were washed twice with PBS, harvested, and lysed with 1 ml of lysis buffer (50 mM Tris-HCl, pH 7.4, 150 mM NaCl, 1% Nonidet P-40, 0.01% SDS and Complete Mini protease inhibitor mixture). After gentle rotation for 1 h, samples were centrifuged for 15 min at 14,000 × *g* and the supernatant was incubated with 50 μl of Dynabeads Protein G for 1 h at 4 °C to remove nonspecific bound proteins. Samples were then incubated with 2 μg of anti-α_2B_-AR antibodies overnight at 4 °C with gentle rotation followed by incubation with 50 μl of Dynabeads Protein G for 4 h. The beads were washed 3 times with lysis buffer without SDS. Immunoprecipitated proteins were eluted with 30 μl of SDS-gel loading buffer and separated by SDS-PAGE. Myc-GGA and HA-α_2B_-AR in the immunoprecipitates were detected by immunoblotting using myc and α_2B_-AR antibodies, respectively.

### GST fusion protein pulldown assays

The GST fusion proteins were expressed in bacteria and purified using a glutathione affinity matrix as described previously^65^,^74^. GST fusion proteins immobilized on the glutathione resin were either used immediately or stored at 4 °C for no longer than 3 days. Each batch of fusion proteins used in experiments was first analyzed by Coomassie Brilliant Blue R250 staining following SDS-PAGE. GST fusion proteins tethered to the glutathione resin were incubated with total cell lysates in 500 μl of binding buffer containing 20 mM Tris-HCl, pH 7.5, 1% NP-40, 140 mM NaCl, 1 mM MgCl_2_ and 0.5% bovine serum albumin at 4 °C for 4–6 h. The resin was washed four times with 0.5 ml of binding buffer and the retained proteins were solubilized in SDS-gel loading buffer and separated by SDS-PAGE. Proteins bound to GST fusion proteins were detected by immunoblotting.

To determine if GGAs could directly interact with α_2B_-AR, GGA1 and GGA2 were tagged with the epitope His at their N-termini in the pET-28 vector and purified by using His SpinTrap kit (GE Healthcare) as described previously^77^. About 1 μg of purified His-GGAs was incubated with GST-ICL3 fusion proteins in 600 μl of binding buffer at 4 °C for 4 h and the retained His-GGAs were measured by immunoblotting using ant-His antibodies.

### Statistical analysis

Differences were evaluated using Student’s *t* test, and *p* < 0.05 was considered as statistically significant. Data are expressed as the mean ± S.E.

## Additional Information

**How to cite this article**: Zhang, M. *et al*. Regulation of α_2B_-Adrenergic Receptor Cell Surface Transport by GGA1 and GGA2. *Sci. Rep.* **6**, 37921; doi: 10.1038/srep37921 (2016).

**Publisher's note:** Springer Nature remains neutral with regard to jurisdictional claims in published maps and institutional affiliations.

## References

[b1] Moore C. A., Milano S. K. & Benovic J. L. Regulation of receptor trafficking by GRKs and arrestins. Annu Rev Physiol 69, 451–482 (2007). 17037978 10.1146/annurev.physiol.69.022405.154712

[b2] Hanyaloglu A. C. & von Zastrow M. Regulation of GPCRs by endocytic membrane trafficking and its potential implications. Annu Rev Pharmacol Toxicol 48, 537–568 (2008). 18184106 10.1146/annurev.pharmtox.48.113006.094830

[b3] Marchese A. & Trejo J. Ubiquitin-dependent regulation of G protein-coupled receptor trafficking and signaling. Cell Signal 25, 707–716 (2013). 23201781 10.1016/j.cellsig.2012.11.024 PMC3593103

[b4] Dong C., Filipeanu C. M., Duvernay M. T. & Wu G. Regulation of G protein-coupled receptor export trafficking. Biochim Biophys Acta 1768, 853–870 (2007). 17074298 10.1016/j.bbamem.2006.09.008 PMC1885203

[b5] Bie B. . Nerve growth factor-regulated emergence of functional delta-opioid receptors. J Neurosci 30, 5617–5628 (2010). 20410114 10.1523/JNEUROSCI.5296-09.2010 PMC2865237

[b6] Yin H. . Rab1 GTPase regulates phenotypic modulation of pulmonary artery smooth muscle cells by mediating the transport of angiotensin II type 1 receptor under hypoxia. Int J Biochem Cell Biol 43, 401–408 (2011). 21095238 10.1016/j.biocel.2010.11.010 PMC3072814

[b7] Filipeanu C. M., Zhou F., Fugetta E. K. & Wu G. Differential regulation of the cell-surface targeting and function of beta- and alpha1-adrenergic receptors by Rab1 GTPase in cardiac myocytes. Mol Pharmacol 69, 1571–1578 (2006). 16461589 10.1124/mol.105.019984

[b8] Binda C. . A G protein-coupled receptor and the intracellular synthase of its agonist functionally cooperate. J Cell Biol 204, 377–393 (2014). 24493589 10.1083/jcb.201304015 PMC3912537

[b9] Tai A. W., Chuang J. Z., Bode C., Wolfrum U. & Sung C. H. Rhodopsin’s carboxy-terminal cytoplasmic tail acts as a membrane receptor for cytoplasmic dynein by binding to the dynein light chain Tctex-1. Cell 97, 877–887 (1999). 10399916 10.1016/s0092-8674(00)80800-4

[b10] McLatchie L. M. . RAMPs regulate the transport and ligand specificity of the calcitonin-receptor-like receptor. Nature 393, 333–339 (1998). 9620797 10.1038/30666

[b11] Colley N. J., Baker E. K., Stamnes M. A. & Zuker C. S. The cyclophilin homolog ninaA is required in the secretory pathway. Cell 67, 255–263 (1991). 1913822 10.1016/0092-8674(91)90177-z

[b12] Dwyer N. D., Troemel E. R., Sengupta P. & Bargmann C. I. Odorant receptor localization to olfactory cilia is mediated by ODR-4, a novel membrane-associated protein. Cell 93, 455–466 (1998). 9590179 10.1016/s0092-8674(00)81173-3

[b13] Ferreira P. A., Nakayama T. A., Pak W. L. & Travis G. H. Cyclophilin-related protein RanBP2 acts as chaperone for red/green opsin. Nature 383, 637–640 (1996). 8857542 10.1038/383637a0

[b14] Chen Y. . GEC1-kappa opioid receptor binding involves hydrophobic interactions: GEC1 has chaperone-like effect. J Biol Chem 284, 1673–1685 (2009). 19001416 10.1074/jbc.M808303200 PMC2615498

[b15] Doly S. . GABA receptor cell-surface export is controlled by an endoplasmic reticulum gatekeeper. Mol Psychiatry (2015). 10.1038/mp.2015.72 PMC4828513 26033241

[b16] Sauvageau E. . CNIH4 interacts with newly synthesized GPCR and controls their export from the endoplasmic reticulum. Traffic 15, 383–400 (2014). 24405750 10.1111/tra.12148

[b17] Zhang X., Dong C., Wu Q. J., Balch W. E. & Wu G. Di-acidic motifs in the membrane-distal C termini modulate the transport of angiotensin II receptors from the endoplasmic reticulum to the cell surface. J Biol Chem 286, 20525–20535 (2011). 21507945 10.1074/jbc.M111.222034 PMC3121450

[b18] Carrel D., Hamon M. & Darmon M. Role of the C-terminal di-leucine motif of 5-HT1A and 5-HT1B serotonin receptors in plasma membrane targeting. J Cell Sci 119, 4276–4284 (2006). 17003106 10.1242/jcs.03189

[b19] Sawyer G. W., Ehlert F. J. & Shults C. A. A conserved motif in the membrane proximal C-terminal tail of human muscarinic m1 acetylcholine receptors affects plasma membrane expression. J Pharmacol Exp Ther 332, 76–86 (2010). 19841475 10.1124/jpet.109.160986 PMC2802481

[b20] Guo Y. & Jose P. A. C-terminal di-leucine motif of dopamine D(1) receptor plays an important role in its plasma membrane trafficking. PLoS One 6, e29204 (2011). 22206002 10.1371/journal.pone.0029204 PMC3242775

[b21] Donnellan P. D., Kimbembe C. C., Reid H. M. & Kinsella B. T. Identification of a novel endoplasmic reticulum export motif within the eighth alpha-helical domain (alpha-H8) of the human prostacyclin receptor. Biochim Biophys Acta 1808, 1202–1218 (2011). 21223948 10.1016/j.bbamem.2011.01.003

[b22] Bermak J. C., Li M., Bullock C. & Zhou Q. Y. Regulation of transport of the dopamine D1 receptor by a new membrane-associated ER protein. Nat Cell Biol 3, 492–498 (2001). 11331877 10.1038/35074561

[b23] Schulein R. . A dileucine sequence and an upstream glutamate residue in the intracellular carboxyl terminus of the vasopressin V2 receptor are essential for cell surface transport in COS.M6 cells. Mol Pharmacol 54, 525–535 (1998). 9730911 10.1124/mol.54.3.525

[b24] Puertollano R., Aguilar R. C., Gorshkova I., Crouch R. J. & Bonifacino J. S. Sorting of mannose 6-phosphate receptors mediated by the GGAs. Science 292, 1712–1716 (2001). 11387475 10.1126/science.1060750

[b25] Doray B., Bruns K., Ghosh P. & Kornfeld S. Interaction of the Cation-dependent Mannose 6-Phosphate Receptor with GGA Proteins. J. Biol. Chem. 277, 18477–18482 (2002). 11886874 10.1074/jbc.M201879200

[b26] Zhu Y., Doray B., Poussu A., Lehto V. P. & Kornfeld S. Binding of GGA2 to the lysosomal enzyme sorting motif of the mannose 6-phosphate receptor. Science 292, 1716–1718 (2001). 11387476 10.1126/science.1060896

[b27] Shiba T. . Structural basis for recognition of acidic-cluster dileucine sequence by GGA1. Nature 415, 937–941 (2002). 11859376 10.1038/415937a

[b28] Misra S., Puertollano R., Kato Y., Bonifacino J. S. & Hurley J. H. Structural basis for acidic-cluster-dileucine sorting-signal recognition by VHS domains. Nature 415, 933–937 (2002). 11859375 10.1038/415933a

[b29] Nielsen M. S. . The sortilin cytoplasmic tail conveys Golgi-endosome transport and binds the VHS domain of the GGA2 sorting protein. EMBO J 20, 2180–2190 (2001). 11331584 10.1093/emboj/20.9.2180 PMC125444

[b30] Takatsu H., Katoh Y., Shiba Y. & Nakayama K. Golgi-localizing, gamma-adaptin ear homology domain, ADP-ribosylation factor-binding (GGA) proteins interact with acidic dileucine sequences within the cytoplasmic domains of sorting receptors through their Vps27p/Hrs/STAM (VHS) domains. J Biol Chem 276, 28541–28545 (2001). 11390366 10.1074/jbc.C100218200

[b31] Nielsen M. S. . Sorting by the cytoplasmic domain of the amyloid precursor protein binding receptor SorLA. Mol Cell Biol 27, 6842–6851 (2007). 17646382 10.1128/MCB.00815-07 PMC2099242

[b32] Boucher R. . Intracellular trafficking of LRP9 is dependent on two acidic cluster/dileucine motifs. Histochem Cell Biol 130, 315–327 (2008). 18461348 10.1007/s00418-008-0436-5

[b33] Doray B., Knisely J. M., Wartman L., Bu G. & Kornfeld S. Identification of acidic dileucine signals in LRP9 that interact with both GGAs and AP-1/AP-2. Traffic 9, 1551–1562 (2008). 18627575 10.1111/j.1600-0854.2008.00786.x PMC2892795

[b34] Jacobsen L. . The sorLA cytoplasmic domain interacts with GGA1 and -2 and defines minimum requirements for GGA binding. FEBS Lett 511, 155–158 (2002). 11821067 10.1016/s0014-5793(01)03299-9

[b35] Ghosh P., Griffith J., Geuze H. J. & Kornfeld S. Mammalian GGAs act together to sort mannose 6-phosphate receptors. J Cell Biol 163, 755–766 (2003). 14638859 10.1083/jcb.200308038 PMC2173681

[b36] Doray B., Ghosh P., Griffith J., Geuze H. J. & Kornfeld S. Cooperation of GGAs and AP-1 in packaging MPRs at the trans-Golgi network. Science 297, 1700–1703 (2002). 12215646 10.1126/science.1075327

[b37] Dell’Angelica E. C. . GGAs: a family of ADP ribosylation factor-binding proteins related to adaptors and associated with the Golgi complex. J Cell Biol 149, 81–94 (2000). 10747089 10.1083/jcb.149.1.81 PMC2175099

[b38] Puertollano R., Randazzo P. A., Presley J. F., Hartnell L. M. & Bonifacino J. S. The GGAs promote ARF-dependent recruitment of clathrin to the TGN. Cell 105, 93–102 (2001). 11301005 10.1016/s0092-8674(01)00299-9

[b39] Shiba T. . Molecular mechanism of membrane recruitment of GGA by ARF in lysosomal protein transport. Nat Struct Biol 10, 386–393 (2003). 12679809 10.1038/nsb920

[b40] Bonifacino J. S. & Traub L. M. Signals for sorting of transmembrane proteins to endosomes and lysosomes. Annu Rev Biochem 72, 395–447 (2003). 12651740 10.1146/annurev.biochem.72.121801.161800

[b41] Wasiak S. . Enthoprotin: a novel clathrin-associated protein identified through subcellular proteomics. J Cell Biol 158, 855–862 (2002). 12213833 10.1083/jcb.200205078 PMC2173151

[b42] Kalthoff C., Groos S., Kohl R., Mahrhold S. & Ungewickell E. J. Clint: a novel clathrin-binding ENTH-domain protein at the Golgi. Mol Biol Cell 13, 4060–4073 (2002). 12429846 10.1091/mbc.E02-03-0171 PMC133614

[b43] Hirst J., Motley A., Harasaki K., Peak Chew S. Y. & Robinson M. S. EpsinR: an ENTH domain-containing protein that interacts with AP-1. Mol Biol Cell 14, 625–641 (2003). 12589059 10.1091/mbc.E02-09-0552 PMC149997

[b44] Mills I. G. . EpsinR: an AP1/clathrin interacting protein involved in vesicle trafficking. J Cell Biol 160, 213–222 (2003). 12538641 10.1083/jcb.200208023 PMC2172650

[b45] Page L. J., Sowerby P. J., Lui W. W. & Robinson M. S. Gamma-synergin: an EH domain-containing protein that interacts with gamma-adaptin. J Cell Biol 146, 993–1004 (1999). 10477754 10.1083/jcb.146.5.993 PMC2169493

[b46] Lui W. W. . Binding partners for the COOH-terminal appendage domains of the GGAs and gamma-adaptin. Mol Biol Cell 14, 2385–2398 (2003). 12808037 10.1091/mbc.E02-11-0735 PMC194887

[b47] Zhang M. . GGA3 Interacts with a G Protein-Coupled Receptor and Modulates Its Cell Surface Export. Mol Cell Biol 36, 1152–1163 (2016). 26811329 10.1128/MCB.00009-16 PMC4800796

[b48] Ang A. L. . Recycling endosomes can serve as intermediates during transport from the Golgi to the plasma membrane of MDCK cells. J. Cell Biol. 167, 531–543 (2004). 15534004 10.1083/jcb.200408165 PMC2172492

[b49] Bertuccio C. A. . Anterograde trafficking of KCa3.1 in polarized epithelia is Rab1- and Rab8-dependent and recycling endosome-independent. PLoS One 9, e92013 (2014). 24632741 10.1371/journal.pone.0092013 PMC3954861

[b50] Parachoniak C. A., Luo Y., Abella J. V., Keen J. H. & Park M. GGA3 functions as a switch to promote Met receptor recycling, essential for sustained ERK and cell migration. Dev Cell 20, 751–763 (2011). 21664574 10.1016/j.devcel.2011.05.007 PMC3115551

[b51] Bonifacino J. S. The GGA proteins: adaptors on the move. Nat Rev Mol Cell Biol 5, 23–32 (2004). 14708007 10.1038/nrm1279

[b52] Wu G. . Identification of Gbetagamma binding sites in the third intracellular loop of the M(3)-muscarinic receptor and their role in receptor regulation. J Biol Chem 275, 9026–9034 (2000). 10722752 10.1074/jbc.275.12.9026

[b53] Wu G., Benovic J. L., Hildebrandt J. D. & Lanier S. M. Receptor docking sites for G-protein betagamma subunits. Implications for signal regulation. J Biol Chem 273, 7197–7200 (1998). 9516410 10.1074/jbc.273.13.7197

[b54] Bockaert J., Perroy J., Becamel C., Marin P. & Fagni L. GPCR interacting proteins (GIPs) in the nervous system: Roles in physiology and pathologies. Annu Rev Pharmacol Toxicol 50, 89–109 (2010). 20055699 10.1146/annurev.pharmtox.010909.105705

[b55] Wu G., Krupnick J. G., Benovic J. L. & Lanier S. M. Interaction of arrestins with intracellular domains of muscarinic and alpha2-adrenergic receptors. J Biol Chem 272, 17836–17842 (1997). 9211939 10.1074/jbc.272.28.17836

[b56] Wang Q. & Limbird L. E. Regulation of alpha2AR trafficking and signaling by interacting proteins. Biochem Pharmacol 73, 1135–1145 (2007). 17229402 10.1016/j.bcp.2006.12.024 PMC1885238

[b57] Pao C. S. & Benovic J. L. Structure/function analysis of alpha2A-adrenergic receptor interaction with G protein-coupled receptor kinase 2. J Biol Chem 280, 11052–11058 (2005). 15653687 10.1074/jbc.M412996200

[b58] Wang X. . Spinophilin regulates Ca2+ signalling by binding the N-terminal domain of RGS2 and the third intracellular loop of G-protein-coupled receptors. Nat Cell Biol 7, 405–411 (2005). 15793568 10.1038/ncb1237

[b59] Bernstein L. S. . RGS2 binds directly and selectively to the M1 muscarinic acetylcholine receptor third intracellular loop to modulate Gq/11alpha signaling. J Biol Chem 279, 21248–21256 (2004). 14976183 10.1074/jbc.M312407200

[b60] DeGraff J. L., Gurevich V. V. & Benovic J. L. The third intracellular loop of alpha 2-adrenergic receptors determines subtype specificity of arrestin interaction. J Biol Chem 277, 43247–43252 (2002). 12205092 10.1074/jbc.M207495200

[b61] Wang Q. . Spinophilin blocks arrestin actions *in vitro* and *in vivo* at G protein-coupled receptors. Science 304, 1940–1944 (2004). 15218143 10.1126/science.1098274

[b62] Richman J. G. . Agonist-regulated Interaction between alpha2-adrenergic receptors and spinophilin. J Biol Chem 276, 15003–15008 (2001). 11154706 10.1074/jbc.M011679200

[b63] Prezeau L., Richman J. G., Edwards S. W. & Limbird L. E. The zeta isoform of 14-3-3 proteins interacts with the third intracellular loop of different alpha2-adrenergic receptor subtypes. J Biol Chem 274, 13462–13469 (1999). 10224112 10.1074/jbc.274.19.13462

[b64] Brady A. E. . Spinophilin stabilizes cell surface expression of alpha 2B-adrenergic receptors. J Biol Chem 278, 32405–32412 (2003). 12738775 10.1074/jbc.M304195200

[b65] Dong C. . A triple arg motif mediates alpha(2B)-adrenergic receptor interaction with Sec24C/D and export. Traffic 13, 857–868 (2012). 22404651 10.1111/j.1600-0854.2012.01351.x PMC3350609

[b66] Weber B., Schaper C., Wang Y., Scholz J. & Bein B. Interaction of the ubiquitin carboxyl terminal esterase L1 with alpha(2)-adrenergic receptors inhibits agonist-mediated p44/42 MAP kinase activation. Cell Signal 21, 1513–1521 (2009). 19477270 10.1016/j.cellsig.2009.05.011

[b67] Dong C., Li C. & Wu G. Regulation of alpha(2B)-adrenergic receptor-mediated extracellular signal-regulated kinase 1/2 (ERK1/2) activation by ADP-ribosylation factor 1. J Biol Chem 286, 43361–43369 (2011). 22025613 10.1074/jbc.M111.267286 PMC3234816

[b68] Wang Q. & Limbird L. E. Regulated interactions of the alpha 2A adrenergic receptor with spinophilin, 14-3-3zeta, and arrestin 3. J Biol Chem 277, 50589–50596 (2002). 12376539 10.1074/jbc.M208503200

[b69] Stojanovic A. & Hwa J. Rhodopsin and retinitis pigmentosa: shedding light on structure and function. Receptors Channels 8, 33–50 (2002). 12402507

[b70] Morello J. P. & Bichet D. G. Nephrogenic diabetes insipidus. Annu Rev Physiol 63, 607–630 (2001). 11181969 10.1146/annurev.physiol.63.1.607

[b71] Conn P. M., Ulloa-Aguirre A., Ito J. & Janovick J. A. G protein-coupled receptor trafficking in health and disease: lessons learned to prepare for therapeutic mutant rescue *in vivo*. Pharmacol Rev 59, 225–250 (2007). 17878512 10.1124/pr.59.3.2

[b72] Wang G. & Wu G. Small GTPase regulation of GPCR anterograde trafficking. Trends Pharmacol Sci 33, 28–34 (2012). 22015208 10.1016/j.tips.2011.09.002 PMC3259232

[b73] Wu G., Zhao G. & He Y. Distinct pathways for the trafficking of angiotensin II and adrenergic receptors from the endoplasmic reticulum to the cell surface: Rab1-independent transport of a G protein-coupled receptor. J Biol Chem 278, 47062–47069 (2003). 12970354 10.1074/jbc.M305707200

[b74] Li C., Fan Y., Lan T. H., Lambert N. A. & Wu G. Rab26 Modulates the Cell Surface Transport of alpha2-Adrenergic Receptors from the Golgi. J Biol Chem 287, 42784–42794 (2012). 23105096 10.1074/jbc.M112.410936 PMC3522277

[b75] Duvernay M. T. . A single conserved leucine residue on the first intracellular loop regulates ER export of G protein-coupled receptors. Traffic 10, 552–566 (2009). 19220814 10.1111/j.1600-0854.2009.00890.x PMC2852481

[b76] Dong C. . Rab8 interacts with distinct motifs in alpha2B- and beta2-adrenergic receptors and differentially modulates their transport. J Biol Chem 285, 20369–20380 (2010). 20424170 10.1074/jbc.M109.081521 PMC2888448

[b77] Dong C. . ADP-ribosylation factors modulate the cell surface transport of G protein-coupled receptors. J Pharmacol Exp Ther 333, 174–183 (2010). 20093398 10.1124/jpet.109.161489 PMC2846028

